# The First Cellular Models Based on Frataxin Missense Mutations That Reproduce Spontaneously the Defects Associated with Friedreich Ataxia

**DOI:** 10.1371/journal.pone.0006379

**Published:** 2009-07-24

**Authors:** Nadège Calmels, Stéphane Schmucker, Marie Wattenhofer-Donzé, Alain Martelli, Nadège Vaucamps, Laurence Reutenauer, Nadia Messaddeq, Cécile Bouton, Michel Koenig, Hélène Puccio

**Affiliations:** 1 IGBMC (Institut de Génétique et de Biologie Moléculaire et Cellulaire), Illkirch, France; 2 Inserm, U596, Illkirch, France; 3 CNRS, UMR7104, Illkirch, France; 4 Université de Strasbourg, Strasbourg, France; 5 Collège de France, Chaire de génétique humaine, Illkirch, France; 6 Institut de Chimie des Substance Naturelles, CNRS, Gif-sur-Yvette, France; University Medical Center Groningen, Netherlands

## Abstract

**Background:**

Friedreich ataxia (FRDA), the most common form of recessive ataxia, is due to reduced levels of frataxin, a highly conserved mitochondrial iron-chaperone involved in iron-sulfur cluster (ISC) biogenesis. Most patients are homozygous for a (GAA)_n_ expansion within the first intron of the frataxin gene. A few patients, either with typical or atypical clinical presentation, are compound heterozygous for the GAA expansion and a micromutation.

**Methodology:**

We have developed a new strategy to generate murine cellular models for FRDA: cell lines carrying a frataxin conditional allele were used in combination with an EGFP-Cre recombinase to create murine cellular models depleted for endogenous frataxin and expressing missense-mutated human frataxin. We showed that complete absence of murine frataxin in fibroblasts inhibits cell division and leads to cell death. This lethal phenotype was rescued through transgenic expression of human wild type as well as mutant (hFXN^G130V^ and hFXN^I154F^) frataxin. Interestingly, cells expressing the mutated frataxin presented a FRDA-like biochemical phenotype. Though both mutations affected mitochondrial ISC enzymes activities and mitochondria ultrastructure, the hFXN^I154F^ mutant presented a more severe phenotype with affected cytosolic and nuclear ISC enzyme activities, mitochondrial iron accumulation and an increased sensitivity to oxidative stress. The differential phenotype correlates with disease severity observed in FRDA patients.

**Conclusions:**

These new cellular models, which are the first to spontaneously reproduce all the biochemical phenotypes associated with FRDA, are important tools to gain new insights into the *in vivo* consequences of pathological missense mutations as well as for large-scale pharmacological screening aimed at compensating frataxin deficiency.

## Introduction

Friedreich ataxia (FRDA), the most common hereditary ataxia, is an autosomal recessive neurodegenerative disease characterized by progressive gait and limb ataxia associated with hypertrophic cardiomyopathy and an increased incidence of diabetes [1], [2]. FRDA is caused by reduced expression of the mitochondrial protein frataxin [3]. The physiopathological consequences of frataxin deficiency are a severe disruption of Fe-S cluster biosynthesis, mitochondrial iron overload coupled to cellular iron dysregulation, and possibly an increased sensitivity to oxidative stress. Frataxin is a highly conserved protein which has been suggested to participate in a variety of pathways associated with cellular iron homeostasis [4]. However, only its essential role as a mitochondrial iron-chaperone for iron-sulfur cluster (ISC) biogenesis is widely accepted. Indeed, frataxin deficiency in human, mouse and yeast leads to severe alteration of mitochondrial and extramitochondrial ISC proteins [5]–[9]. Very recently, the bacterial frataxin has been proposed to be an iron sensor that act as a regulator of Fe-S cluster formation [10].

The most common mutation leading to FRDA is a (GAA)_n_ triplet repeat expansion within the first intron of the frataxin gene [3]. 96% of patients carry two expanded alleles which lead to a partial transcriptional silencing, either through the formation of a triple helix which interferes with transcriptional elongation [11] or epigenetic changes leading to heterochromatin formation thereby impairing gene transcription [12]. This results in a strongly reduced frataxin protein expression in all tissues. This partial silencing is of importance as in multicellular eukaryotes, frataxin is essential for embryonic development. Indeed, complete frataxin deletion leads to early embryonic lethality in plants and mice [13], [14], to L2/L3 larval stage arrest in *C. elegans* [15], and to reduced larval viability and metamorphosis failure by systemic silencing in the *Drosophila* [16], [17].

A small but significant number of FRDA patients (4%) are compound heterozygous for the (GAA)_n_ expansion and a micromutation [18], [19]. Out of the 40 pathogenic sequence variants reported in frataxin, at least 15 missense point mutations have been described. There are a few prevalent mutations that result either in classical FRDA phenotype (I154F and W155R) or an atypical clinical presentation (G130V). Isoleucine 154 is a hydrophobic residue involved in the protein core [20], [21]. The I154F mutation has been described in six patients from four families from South Italy [3], [18], [19], [22]. The clinical presentation of these patients is indistinguable from that of patients homozygous for the expansion. The G>T transversion at nucleotide 389 leading to the G130V replacement is the most frequent missense mutation in FRDA, found in more than ten Caucasian patients from five families ([18], [23]–[25] and MK unpublished data). Although the age of onset is within the first ten years in patients carrying the G130V mutations, disease progression is remarkably slow. The absence of dysarthria and the persistence of knee jerks are consistent findings in these patients. A more spastic than ataxic gait has also been described [18], [25].

Mammalian cell models are important to study the molecular mechanisms of disease and are powerful tools for large-scale therapeutical screening approaches. As patient's cell line (fibroblasts or lymphocytes) do not express consistently the biochemical phenotypes associated with FRDA under basal cultured conditions, RNA interference [26]–[30] strategies have been developed to reproduce partial frataxin deficiency in human and murine cell lines. A few models exhibit some of the biochemical features associated with FRDA, notably the deficiency in ISC biosynthesis and the increased sensitivity to oxidative stress [26], [28], [30], but none reproduce the mitochondrial iron accumulation seen in patients. In the present work, we developed novel cellular models deleted for endogenous murine frataxin and expressing a human frataxin cDNA carrying pathogenic missense mutations resulting in a classical (I154F) and an atypical (G130V) FRDA clinical presentation. The models obtained are the first stable cellular models that reproduce spontaneously all the biochemical phenotype associated with FRDA.

## Results

### Complete frataxin deletion in murine fibroblasts is not viable and can be rescued by transgenic expression of mFxn

**Figure 1:**
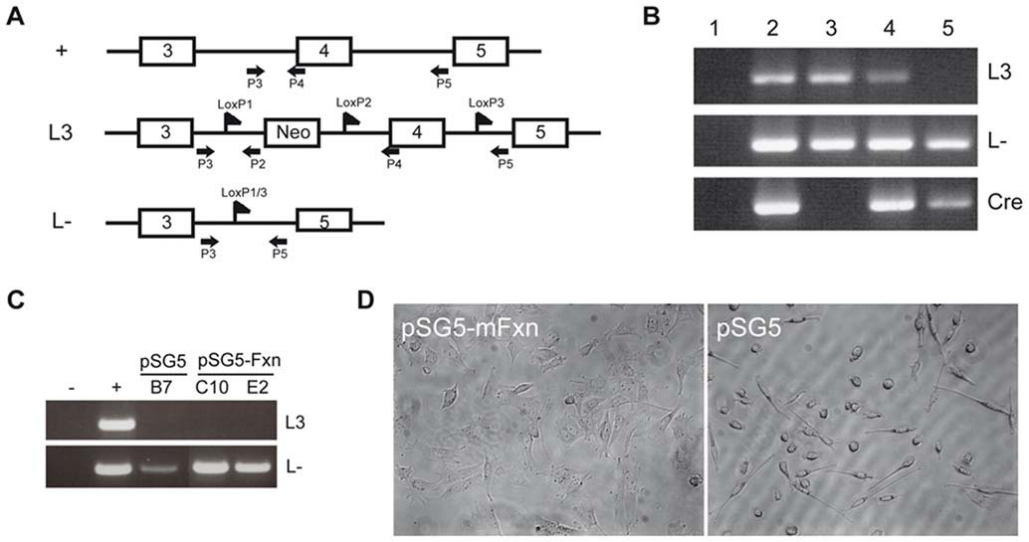
Frataxin is essential for division and survival of murine fibroblasts. A. Schematic representation of wild-type murine frataxin allele (Frda^+^), the loxP-flanked Frda exon 4 allele (Frda^L3^) and the Cre-mediated deleted allele (Frda^L-^). Primers used for genotyping are represented by arrows [7]. B. Genotyping on heterozygous Frda^L3/L-^ cells before (lane 3) and after (lanes 4 and 5) pEGFP-Cre transfection and sort. Adherent living cells display a Frda ^L3/L-^, Cre genotype (lane 4) whereas floating cells are completely deleted for frataxin (Frda^L-/L-^) (lane 5). Lane 2: positive controls of each PCR. Lane 1: negative control (no DNA). C. Genotyping of Frda^L3/L-^ clones after pEGFP-Cre transfection and sort. Results from one clone transfected with an empty vector (pSG5: Frda^L3/L-; empty^, clone B7) and two clones overexpressing murine frataxin (pSG5-mFxn: Frda^L3/L-; mFxn^, clones C10 and E2). First lane: no DNA (−). Second lane: positive controls of each PCR (+). D. Phase contrast microscopy on rescued Frda^L3/L-^ clones after pEGFP-Cre transfection and sort. pSG5-mFxn: normal morphology of a murine frataxin overexpressing clone (Frda^L3/L-; mFxn^) and deleted for endogenous frataxin. pSG5: cells from a clone transfected with the empty vector (Frda^L3/L-; empty^). Note the presence of many elongated or round unhealthy cells. Phase contrast (20× magnification), cells plated in 96-well plastic plates.

Since fibroblasts of patients with mutations directly affecting the respiratory chain are viable in cell culture, irrespective of viability in the animal, we sought to obtain a FRDA cell model by frataxin deletion in mouse fibroblasts. To this end, an immortalized fibroblast cell line compound heterozygous for the mouse frataxin conditional allele and the deleted allele (Frda^L3/L-^) [7] (Fig. 1A) was transfected with a fluorescent recombinase (pEGFP-Cre) and sorted by FACS 48 hours after transfection. Most sorted cells stopped dividing after 2–3 days in culture, became round, to end up floating in the culture medium after 7–10 days in culture. The genotype of the total cellular population demonstrated that not all adherent cells were deleted for frataxin (Fig. 1B, lane 4), while the floating cells harvested from the supernatant presented a clear Frda^L-/L-^ genotype (Fig. 1B, lane 5) suggesting that complete frataxin deletion is lethal in fibroblast.

To confirm that frataxin deletion is the cause of cell detachment and subsequent death, we determined whether transgenic expression of murine frataxin (mFxn) could rescue the phenotype. The mFxn cDNA (pSG5-mFxn) was stably expressed into Frda^L3/L-^ cell line (Frda^L3/L-; mFxn^). Western blot analysis confirmed a three fold increase in frataxin protein compared to endogenous levels with no effect on the general morphology of the cell lines (data not shown). pEGFP-Cre transfection was then performed on both Frda^L3/L-; mFxn^ and control cell lines. Transfected cells were sorted by FACS one cell per well and the clones were cultured for seven to ten days. Nineteen clones overexpressing mouse frataxin (Frda^L3/L-; mFxn^) presented a complete deletion of the endogenous frataxin gene (Fig. 1C, clones C10 and E2). All clones displayed normal morphology (Fig. 1D, left panel) and growth, demonstrating that transgenic expression of frataxin rescues the lethality associated with complete deficiency of endogenous frataxin. Frataxin protein overexpression was confirmed in several deleted clones (Fig. S1). Only five clones were obtained from the cells transfected with the empty vector (Frda^L3/L-;pSG5^). These clones exhibited an abnormal morphology with a few dozen spindle-shaped fibroblasts, which became round and barely attached to the plate after 5–7 days of culture (Fig. 1D, right panel). These clones clearly stopped dividing. Genotyping of the five unhealthy clones performed on the total cell lysates (DNA extraction was avoided due to the small amount of cells) was successful in only three cases: the conditional Frda^L3^ allele was converted into the deleted Frda^L-^ allele in all three clones (Fig. 1C, clone B7). Frataxin protein deficiency could not be evaluated by western blot as too few cells were available. In electron microscopy studies, apart from the retraction of the plasma membrane leading to an organelle dense cytoplasm, no structural anomaly in the mitochondria nor sign of necrosis or apoptosis was detected in Frda^L3/L-; pSG5^ sorted clones (Fig. S2). Together, these results confirm the deleterious consequence of frataxin deficiency on cell proliferation and survival.

### Transgenic expression of pathological hFXN mutants partially rescues the endogenous frataxin deficiency

**Figure 2:**
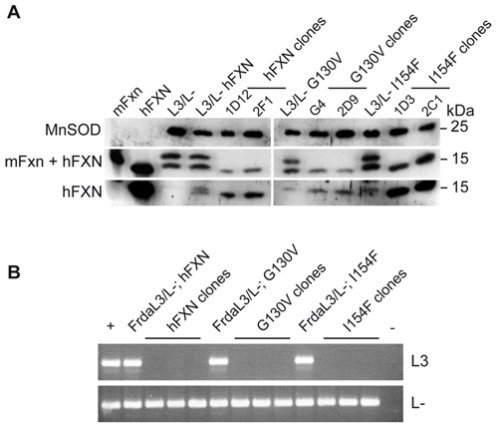
Murine frataxin (mFxn) deficiency and human frataxin (hFXN) expression in the missense mutants. A. Western Blot analysis on mitochondria-enriched fractions using anti-frataxin (R1270 and 1G2) and anti-MnSOD antibodies. hFXN and mFxn lanes correspond to COS cells transfected with wild-type human and murine frataxin expression vector, respectively. The R1270 antibody was designed against murine frataxin but cross-reacts with the human protein. The 1G2 antibody recognizes only human frataxin. Murine and human frataxin were separated using a long SDS-PAGE gel. Note that murine frataxin was detected as a two-band signal in *Frda* ^L3/L-^ fibroblasts. B. Genotyping on heterozygous Frda^L3/L-^ cell populations expressing either wild type or mutant (G130V or I154F) hFXN before (Frda^L3/L-; hFXN^; Frda^L3/L-; G130V^; Frda^L3/L-; I154F^) and after (hFXN, G130V and I154F clones, three different clones for each) pEGFP-Cre transfection and clonal sorting. First lane: positive controls of each PCR (+). Last lane: no DNA (−).

We sought to use this newly developed *ex vivo* system as a novel tool to generate cell models expressing a human frataxin cDNA carrying pathogenic missense mutations. The wild type human frataxin (hFXN) cDNA (pcDNA-hFXN) and both mutant human frataxin cDNA (pcDNA-G130V and pcDNA-I154F) were stably expressed into Frda^L3/L-^ cell line (Frda^L3/L-; hFXN^; Frda^L3/L-; G130V^; Frda^L3/L-; I154F^). Quantitative RT-PCR (not shown) and western blot analysis confirmed the expression and correct mitochondrial targeting and maturation of the three frataxin constructs (Fig. 2A, lanes 4, 7 and 10).

To delete the endogenous murine frataxin, pEGFP-Cre transfection and clonal sorting was performed. After the sort, 13, 6 and 3 clones were obtained for the Frda^L3/L-; hFXN^, Frda^L3/L-; G130V^ and Frda^L3/L-; I154F^ cell lines, respectively. As a control, similar experiments with an Frda^L3/L-^ cell line stably transfected with a truncated form of hFXN (exon 1 and 2 only) resulted in no clone. Genotyping demonstrated a complete deletion of the endogenous murine frataxin gene for each clone, hereafter referred as hFXN, hFXN^G130V^ and hFXN^I154F^ clones (Fig. 2B). Quantitative RT-PCR and western blot analysis confirmed deletion of the endogenous mouse frataxin transcript (data not shown) and absence of the murine protein (Fig. 2A) in each clone. Western blot analysis of mitochondria-enriched fractions of the different clones demonstrated proper maturation of the hFXN^G130V^ and hFXN^I154F^ proteins (Fig. 2A, lanes 8–9 and 11–12, respectively). Interestingly, all I154F clones showed a high expression level of the hFXN^I154F^ protein compared to the wild-type or hFXN^G130V^ proteins, correlating with a 4–10 fold increased level of the transcript (data not shown). All clones showed sustained growth, demonstrating that each human construct was able to rescue the lethal phenotype.

### New “humanized” cell models for FRDA based on the missense mutations

**Figure 3:**
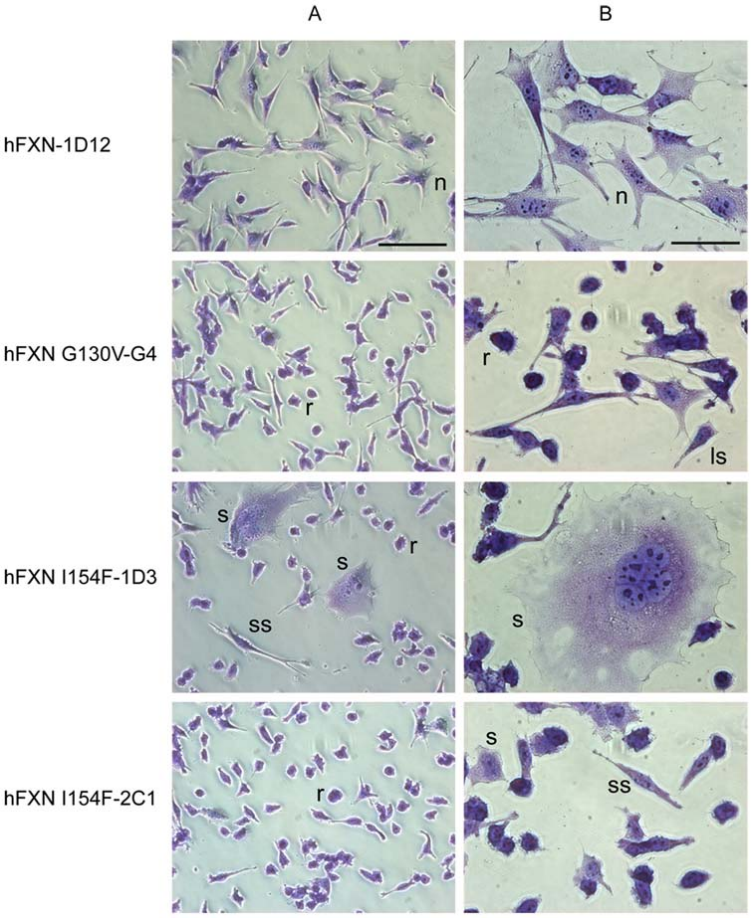
Altered morphology of frataxin mutated clones. Morphology of one normal human frataxin clone (hFXN-1D12), one G130V mutated clone (hFXN^G130V^-G4) and two I154F mutated clones (hFXN^I154F^-1D3 and hFXN^I154F^-2C1) were studied by phase contrast microscopy after crystal violet staining. The scale represents 50 µm in A and 25 µm in B. n, normally spread fibroblast; ls, less spread cell; r, rounded cell; ss, spindle-shaped cell; s, senescent cell.

All isolated clones were viable and proliferated over multiple passages in classical culture conditions. However, compared to the clones expressing wild type hFXN, the hFXN^I154F^ expressing clones repeatedly showed a growth defect, taking a longer time to reach confluence. This growth defect was particularly noticeable after strong dilution of the cell line. Although consistent, the growth defect was not sufficiently severe to cause a significant difference over a 4-day proliferation curve (data not shown). Interestingly, the hFXN^G130V^ expressing clones did not show any growth defect. Furthermore, while the clones expressing the wild type hFXN presented normal fibroblast morphology (Fig. 3, line hFXN-1D12), the hFXN^G130V^ expressing clones displayed a slightly altered morphology with smaller or less spread out cells (Fig. 3, line hFXN^G130V^-G4). In correlation with the growth defect, the hFXN^I154F^ expressing clones displayed a very altered morphology, with numerous small grainy rounded cells with a retracted cytoplasm and some elongated spindle-shaped cells (Fig. 3, lines hFXN^I154F^-1D3 and hFXN^I154F^-2C1). Finally, gigantic cells reminiscent of senescence features were also observed in the three hFXN^I154F^ expressing clones. These cells were spread out and exhibited fractionated nucleus and cytosolic vacuoles (Fig. 3, line I154F-1D3).

**Figure 4:**
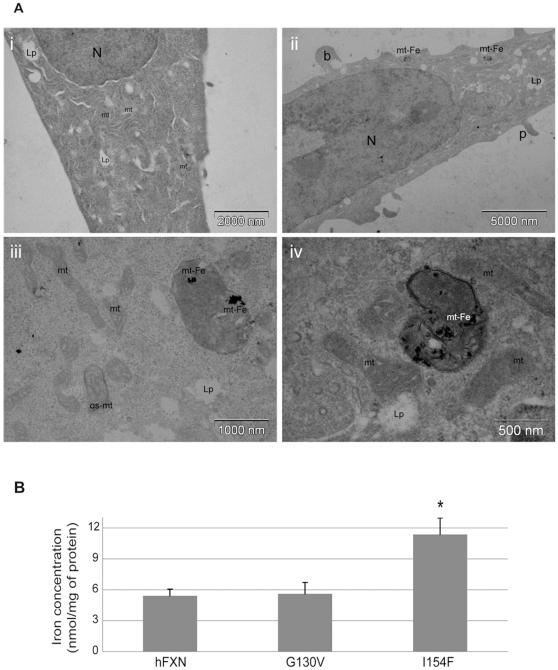
Ultrastructural alterations and iron status in hFXN^G130V^ and hFXN^I154F^ clones. A. Electron microscopy on hFXN (i), and hFXN^I154F^ (ii–iv) clones. b, plasma membrane blebbing; g-mt, giant mitochondria; Lp, lipid droplet; mt, mitochondria; mt-Fe, electron-dense intramitochondrial deposits; N, nucleus; os-mt, onion-shaped mitochondria; p, pseudopodia; rer, rough endoplasmic reticulum. B. Determination of iron concentration. Total iron concentration of mitochondrial-enriched fractions of the different clones was assessed spectrophotometrically using bathophenanthroline as described in material and methods. Results are given as mean of nmol of iron per mg of protein in each fraction + SD. * p<0.01.

The ultrastructural analysis by electron microscopy of both hFXN^G130V^ and hFXN^I154F^ clones revealed strongly altered mitochondrial morphology with little damage to other components of the cell. At the cellular level, the hFXN^G130V^ and hFXN^I154F^ clones presented cytoplasmic blebbing, an ultrastructural sign of reversible cell injury, and increased pseudopodia (Figs. 4A and S3). While abnormal mitochondria were rare events in the wild-type hFXN clones, several alterations of the mitochondria were observed in the hFXN^G130V^ and hFXN^I154F^ clones. Only few mitochondria appeared intact with normal cristae membranes, and many showed spherical shape, either lacking cristae membranes, exhibiting central tubular cristae or onion-shaped inner membrane structures (Fig. 4A and S3). Interestingly, a significant number of giant disorganised mitochondria, derived from mitochondrial fusion, was observed in the hFXN^I154F^ clones. It is important to note that some intramitochondrial electron-dense deposits consistent with iron accumulation were detected in both clones, often associated to the mitochondrial membranes (Fig. 4A). While small deposits were detected in only 10% of the hFXN^G130V^ cells, more than 50% of the cells in the hFXN^I154F^ clones presented several mitochondria with large intramitochondrial deposits. Direct measurements of the iron content of mitochondrial-enriched fraction of the different cell lines demonstrated that the hFXN^I154F^ clones present a two fold iron accumulation compared to the hFXN clones (Fig. 4B). By atomic absorption spectroscopy, the iron content of the soluble mitochondrial fraction showed a slight but not significant increased in iron content (Fig. S4). However, the iron content of the insoluble membrane pellet of the hFXN^I154F^ clones was 2.3 fold higher compared to the hFXN clones (Fig. S4), further supporting that the electron-dense deposits correspond to iron rich aggregates. As a loss of cristae membranes has been described for human cell that are devoid of mitochondrial DNA (rho^0^ cells), we checked the integrity of mitochondrial DNA by long-range PCR. No difference was found between the hFXN and mutant clones (data not shown), indicating that these cells did not loose their mitochondrial DNA.

**Figure 5:**
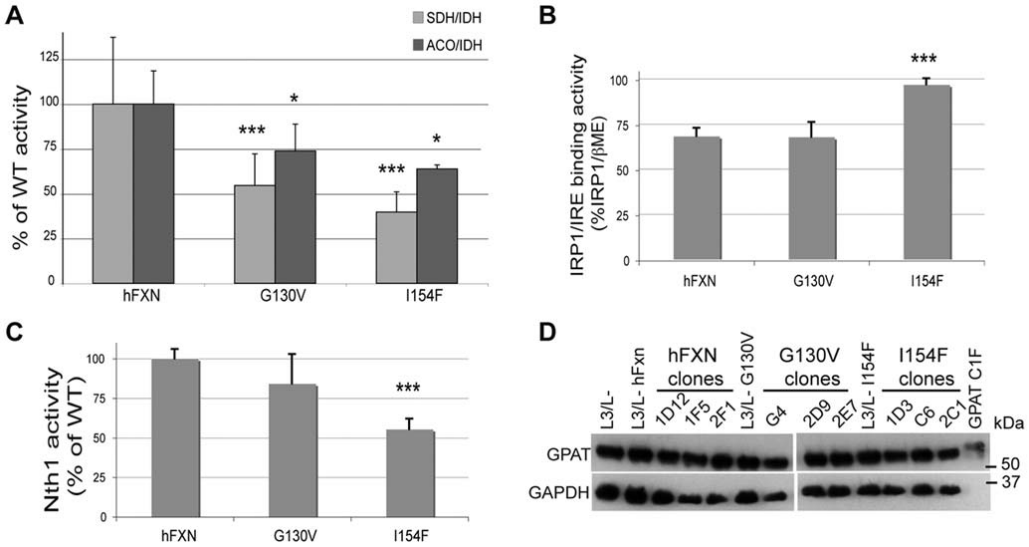
Biochemical measurements of ISC enzyme activities in wild-type hFXN, mutant hFXN^G130V^ and hFXN^I154F^ clones. A. Complex II of respiratory chain (Succinate Quinone DCPIP Reductase, SQDR; white bars) and aconitases (grey bars) specific activities were standardized to isocitrate dehydrogenase (IDH) specific activity and expressed as percentage of control activity. B. IRP1/IRE binding activity was measured by EMSA. Signals in the absence and in the presence of 2% of 2-βmercaptoethanol (βME) were quantified to determine the percentage of IRE-binding IRP1 in each clone. C. Nth1 activity was determined by the thymidine glycol nicking activity of crude nuclear extracts of each clone. Results, expressed as percentage of control activity, are given as the mean of the quantified intensity of Nth1 cleaved product signal from the thymidine glycol containing oligo observed on a 20% denaturing polyacrylamide gel. D. Western blot analysis using a specific anti-GPAT antibody. GAPDH was used as a loading control. GPAT C1F represents COS cells expressing a mutated GPAT which cannot be maturated, giving the GPAT precursor migration size. All values were generated by determining the means of two or three independent experiments performed on different cell extracts on at least 3 clones of hFXN, hFXN^G130V^ or hFXN^I154F^. Data are represented as mean + SD. * p<0.05; *** p<0.005.

To assess the consequences of the missense mutations on the ISC-related function of frataxin, the activities of mitochondrial, cytosolic and nuclear ISC containing enzymes known to be decreased in FRDA heart biopsies [5] or FRDA conditional mouse models [7]–[9] were measured. Both hFXN^G130V^ and hFXN^I154F^ clones showed a significant decrease in the mitochondrial succinate dehydrogenase (SDH) activity with a more severe effect in the hFXN^I154F^ mutants compared to the hFXN^G130V^ mutants with 36% and 54% residual SDH activity, respectively (Fig. 5A). The activity of the aconitases was also decreased in both hFXN^G130V^ and hFXN^I154F^ clones, although to a lesser degree than the SDH activity, with 74% and 64% residual aconitase activity, respectively (Fig. 5A). The iron regulatory protein 1 (IRP1) is a bifunctional ISC protein located in the cytosol [31]. In the presence of [4Fe-4S] cluster, it functions as an aconitase. Devoid of its ISC, it binds to specific mRNA stem loop structures called IRE to regulate the expression of proteins involved in iron homeostasis. An increase in the IRE binding activity of IRP1 was measured only in the hFXN^I154F^ clones compared to the parental cells (Fig. 5B). To determine whether there was a deficit in a nuclear ISC protein, we evaluated the activity of Nth1, a [4Fe-4S] glycosylase/AP-lyase involved in the base excision repair of oxidized bases such as thymine glycol (Tg) [32]. A significant decrease in the activity of Nth1was observed specifically in the hFXN^I154F^ clones (Fig. 5C). The change in activity was not due to a transcriptional regulation, as RT-PCR showed no difference in Nth1 expression between the different clones (data not shown). Note that the decrease in the ISC enzymes activities described above occurs spontaneously, without the addition of exogenous stress. Furthermore, the severity of the deficit was variable from one clone to another. Finally, not all ISC enzymes were affected in the hFXN^I154F^ clones. Indeed, glutamine phosphoribosylpyrophosphate aminotranferase (GPAT), an enzyme of purine biosynthesis which requires the incorporation of a [4Fe-4S] cluster to generate the mature enzyme [33], is not affected in both the hFXN^G130V^ and hFXN^I154F^ clones (Fig. 5D), suggesting that some ISC enzymes are less sensitive to frataxin functional impairment.

**Figure 6:**
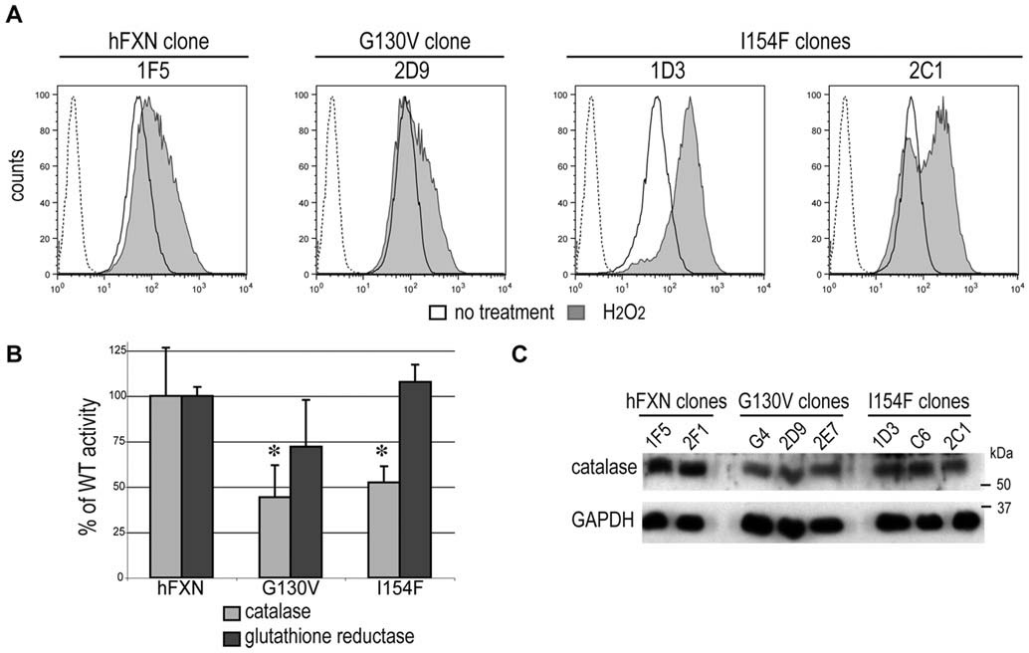
ROS determination and antioxidative enzymes activities in wild-type hFXN, mutant hFXN^G130V^ and hFXN^I154F^ clones. A. FACS analysis of cells incubated with DHR123. The dashed curve represents the autofluorescence of fibroblasts cells without DHR123 treatment. The black curve represents the observed fluorescence in cells in endogenous conditions and the grey curve is the fluorescence induced after hydrogen peroxide incubation (10 µM; 30 min). Experiments were done in triplicate on 3 clones of hFXN, hFXN^G130V^ and hFXN^I154F^. Results show one representative diagram from one clone for hFXN and hFXN^G130V^, and 2 clones for hFXN^I154F^. B. Enzymatic actvities of catalase (grey bars) and glutathione reductase (black bars) were determined spectrophotometrically. Activities are presented as the percentage of WT hFXN clones activity. Data are represented as mean + SD. * p<0.05. C. Catalase protein was detected using an anti-catalase antibody and compared to GAPDH as a loading control.

A recognized characteristic of frataxin deficiency is the increased susceptibility to induction of endogenous or exogenous oxidative stress in FRDA patient cells, and in yeast, *C.elegans* and *Drosophila* models [4], [34]. To investigate the oxidative status of the newly developed cell models, we monitored the presence of intracellular ROS (peroxides and peroxynitrite anion) using the dihydrorhodamine 123 probe. Comparison of oxidation-induced fluorescence of the probe by FACS between hFXN, hFXN^G130V^ and hFXN^I154F^ clones revealed no difference in the production of ROS in normal culture conditions (Fig. 6A). However, the hFXN^I154F^ clones were more sensitive to exogenous stress, as a treatment with 10 µM hydrogen peroxide caused a drastic change in fluorescence level in hFXN^I154F^ clones compared to moderate effects on hFXN and hFXN^G130V^ clones (Fig. 6A). Furthermore, the hFXN^I154F^-2C1 clone had a lower susceptibility to stress than the hFXN^I154F^-1D3 clone since the fluorescence peak in the later was fully shifted. This hFXN^I154F^-2C1 clone appeared globally less affected than the two others hFXN^I154F^ clones (1D3 and C6). Interestingly, both hFXN^G130V^ and hFXN^I154F^ clones displayed a significantly reduced catalase activity (44% and 51%, respectively, Fig. 6B) that correlated with a decrease in catalase expression at the protein level (Fig. 6C) and at the mRNA level (not shown). On the other hand, no significant change was observed in the activity of glutathione reductase (Fig. 6B). Furthermore, no difference in the mitochondrial superoxide dismutase (Sod2) expression at the protein level (Fig. 2A) or at the RNA level (data not shown) was observed. Overall, these results suggest that the combination of iron deregulation with a deficit in the antioxidant enzyme catalase could explain the susceptibility to exogenous oxidative stress observed for the hFXN^I154F^ clones.

## Discussion

In the present study, we report the first cellular models for FRDA based on pathological missense mutations which spontaneously show all the biochemical features of the human disease. Using a fluorescent Cre recombinase in fibroblasts carrying the conditional frataxin allele, we first demonstrated that complete frataxin deficiency in a fibroblast cell line does not sustain cell division and survival. Both transgenic expression of murine (mFxn) and human frataxin (hFXN) can rescue this lethality. Through this novel *in vitro* system, we generated two humanized cell models by combining the deletion of the endogenous murine conditional frataxin allele with the expression of human frataxin carrying either the G130V or I154F missense mutations found in compound heterozygous FRDA patients. Of interest, the two humanized cell models show different severities in their phenotype, which correlate with the disease severity observed in FRDA patients.

As in mouse models [35], [36], hFXN can fully substitute for mFxn at the cellular level. Indeed, the hFXN clones show no morphological or biochemical alterations. In contrast, although both hFXN^G130V^ and hFXN^I154F^ can rescue the lethality of complete mFxn deficient cells, the sole expression of mutated human frataxin does not fully restore functional mitochondria. Indeed, the hFXN^G130V^ and hFXN^I154F^ mutant clones show degenerating mitochondria with disorganized cristae and electron-dense deposits, and a specific deficit in mitochondrial ISC enzymes activities, which are features characteristic of FRDA. In addition, the hFXN^ I154F^ clones exhibit a deficit in the nuclear ISC cluster DNA glycosylase, Nth1, a dysregulated IRP1 IRE-binding activity, an increased mitochondrial iron content, and an increased susceptibility to exogenous oxidative stress. Interestingly, not all ISC enzymes are affected equivalently by frataxin deficiency. As previously reported in the nervous tissues of conditional frataxin knockout mouse models [7], [37] as well as in Hela cells silenced for frataxin [30], the activity of SDH is more affected than the activity of the mitochondrial aconitase. In addition, GPAT maturation which requires an ISC, is not affected in the more severe hFXN^I154F^ clones. This observation is in agreement with data from the conditional mouse models that suggest a threshold effect of ISC enzyme activities that is both tissue-specific as well as dependent on the disease progression [8]. Indeed, the new “humanized” cell models that we are reporting present a milder ISC enzymes deficiency than the conditional knockout mouse models. These results suggest that the affected tissues in FRDA should not present a deficiency in all ISC enzymes. It will be of interest in the future to uncover which ISC enzymes participate in the pathology of the different tissues.

The “humanized” cell models developed are a valuable addition to the available cellular models for FRDA research. Indeed, in contrast to fibroblasts or lymphocytes cell lines from FRDA patients, which show a large variability and a lack of reproducibility depending on the culture conditons, the biochemical phenotype of the “humanized” cell models is stable over multiple passages. Furthermore, the “humanized” cell models spontaneously exhibit multiple ISC enzymes deficit, while fibroblasts from FRDA patients present an ISC enzymes deficit only under exogenous stress [38], [39]. Moreover, the hFXN^I154F^ clones present a clear 2.3 fold increased in mitochondrial iron, a feature not found in FRDA fibroblasts nor lymphocytes [40]–[42]. A common feature between the FRDA fibroblasts and the hFXN^I154F^ clones is the increased sensitivity to exogenous oxidative stress [38], [42]–[44]. Therefore, as both the G130V and I154F mutant clones display a stable and spontaneous biochemical phenotype and are able to proliferate in classical culture conditions, these new cellular models will be useful for functional-based large-scale drug screening.

The pathological missense mutations affect murine fibroblasts in a manner that correlates with the disease severity in compound heterozygotes. The hFXN^G130V^ mutant appears more efficient to rescue lethality than the hFXN^I154F^ mutant although neither clone completely rescues the phenotype. The global morphological alterations as well as the ISC enzymes deficits are less severe in the hFXN^G130V^ clones. Furthermore, the ultrastructural abnormalities in mitochondria as well as intramitochondrial iron deposits were more frequent in the hFXN^I154F^ mutants and a growth defect was only observed in this more severe model. We observed a high expression level of the hFXN^I154F^ transgene compared to the wild-type or hFXN^G130V^ mutant, pointing to a severe functional impairment of the hFXN^I154F^ protein. Indeed, during the clonal selection, only cells expressing high copy number of this severe mutant were able to undergo cell proliferation.

The increased susceptibility to exogenous oxidative stress of the hFXN^I154F^ clones probably arises from strong iron homeostasis dysregulation in conjunction with a decreased activity of the antioxidant enzyme, catalase. Recently, Anderson *et al.* have shown that ectopic expression of enzymes that scavenge H_2_0_2_ (peroxisomal and mitochondrial catalases and a mitochondrial peroxiredoxin) in a *Drosophila* model deficient for frataxin suppresses some of the deleterious phenotypes associated with frataxin deficiency, while overexpression of enzymes that scavenge superoxides (SOD1 and SOD2) have no effect [45]. The present results further support the notion that the increased susceptibility of frataxin deficient cells is linked to an impaired capacity to detoxify H_2_0_2_, although the sole impairment of catalase is not sufficient to result in increased susceptibility (hFXN^G130V^ clones). Finally, it would be of interest to test the potential effect of catalase mimetics on the hFXN^I154F^ cellular model. Indeed, a recent study demonstrate that a catalase mimetic can restore the responsiveness of the Nrf2-dependent signaling pathway to induced oxidative stress in frataxin-depleted cells [40].

The genotype-phenotype correlation observed in our cell models is not fully explained by structural and functional studies. A comparative study of the protein dynamics of human frataxin variants has revealed that although destabilized, both hFXN^G130V^ and hFXN^I154F^ mutant proteins should be properly folded in physiological conditions [46]. However, the two frataxin variants show different iron binding properties. While the G130V mutation decreases iron affinity of the protein, the hFXN^I154F^ mutant protein precipitates in presence of iron [46], [47]. The models that we have developed constitute powerful tools to study the *in vivo* properties of the two mutant proteins, and to test whether the differential *in vitro* iron binding property of the mutant proteins is relevant *in vivo*.

In addition, our results show for the first time that complete frataxin deficiency in a fibroblast cell line does not sustain cell division and survival. The deficit in the activity of the respiratory chain complexes alone may not explain the severity of the phenotype, as fibroblasts carrying mitochondrial respiratory chain deficiency are viable in culture. It is therefore tempting to speculate that the phenotype associated with complete deficiency of frataxin in fibroblast is linked to the deficit of alternative essential ISC proteins, such as the eukaryotic primase [48], necessary for DNA replication, and the ABCE1 protein [49], essential for translation initiation and ribosome maturation. Further investigations to test these hypotheses are however difficult due to the paucity of these frataxin deficient cells.

While some pharmacological compounds have shown some promising results in clinical trials in providing protection on certain aspects of the disease, there is still no effective therapy for FRDA. A valuable therapeutic approach would be frataxin replacement either by gene therapy or by protein replacement using protein transduction domains. We believe that the *in vitro* system that we have generated will provide a powerful tool to functionally evaluate frataxin protein delivery methods or frataxin replacement therapeutic strategies.

## Materials and Methods

### Plasmid constructs, molecular analysis, and mitochondrial-enriched fraction

The complete coding sequence (nucleotide sequence 1–624) xof mouse frataxin (mFxn) was subcloned from the pTL1 vector [50] to the pSG5-Puro-Flag vector (modified from pSG5 vector, Stratagene) by enzymatic restriction. The complete coding sequence of human frataxin (hFXN) wild-type (nucleotide sequence 1–632), C-terminal truncated form encoding exons 1 and 2 only (nucleotide sequence 1–263) and full length hFXN carrying the G130V and I154F missense mutations were subcloned from the pTL1 vectors [50], [51] to the pcDNA 3.1/Zeo (Invitrogen) vector either by enzymatic restriction or PCR. The cDNA encoding bacteriophage P1 Cre recombinase was cloned between the *BglII* and *EcoRI* restriction sites of pEGFP-C2 (Clontech), in frame with and 3′ to the EGFP cDNA. All constructs were checked by sequencing. Genotyping was performed as previously described [7]. Quantitative RT-PCR of mouse frataxin, hprt, Sod1, Sod2, and Nth1 transcripts were performed on a LightCycler 480 (Roche) as previously described [7], [8], [9]. Amplification of the human frataxin-specific transcript and the mouse catalase transcript was performed using primers described in Text S1. The integrity of mitochondrial DNA was checked by large-fragment PCR using primers (3278 and 13337) as described [52]. Total protein extract and mitochondria-enriched fractions obtained by digitonin treatment [53] were analyzed on SDS-Glycine-PAGE (10–13.5% acrylamide). Western blot and antibody dilution were as previously described [8].

### Cell culture, transfection, and FACS sorting

Murine fibroblast cultures from frataxin heterozygous (*Frda* ^L3/L-^) mice (Fig. 1A) [7] were established using the primary-explant technique [54], and immortalized by transfection with a Large Antigen T construct. All transfections were performed using the Fugene 6 Transfection Reagent kit (Roche). Stably transfected fibroblasts were grown in DMEM media (Sigma, Saint Louis, Missouri) with 10% fetal calf serum and 50 µg/ml gentamycin, supplemented either with puromycin (5 µg/ml) or zeocin (250 µg/ml). Antibiotic selection was maintained for one month to obtain stable cell lines.

Cell sorting was performed 48 hrs after transfection. Trypsinised cells were resuspended in PBS and filtered through a 50 µm sterile mesh (BD Biosciences), and were then sorted for EGFP expression on a FACSDiVa Vantage (Becton-Dickinson). EGFP positive cells were either isolated one cell per well in 96-well plates filled with conditional medium or collected all together (population).

### Phase contrast, crystal violet staining, and electron microscopy

Cells were fixed with methanol and stained with crystal violet 0.1%. Living cell cultures and stained cells were observed using a Leica DMLB inverted microscope with Hoffman contrast (LMC) (10× magnification) or phase contrast (20× magnification) and photographs were taken by a CoolSnap camera. For electron microscopy, cellular clones were fixed in a freshly made mixture of 2.5% paraformaldehyde and 2.5% glutaraldehyde in cacodylate buffer (0.1 M, pH 7.2), rinsed in cacodylate buffer, postfixed in 1% osmium tetroxide in 0.1 M cacodylate buffer for 1 hour at 4°C, dehydrated, and embedded in Epon. Ultrathin sections were cut at 70 nm and contrasted with uranyl acetate and lead citrate and examined with a Morgagni 268D electron microscope. Three to five clones were analyzed from each cell line.

### Biochemical analyses

The activities of the succinate dehydrogenase (SDH), aconitases and isocitrate dehydrogenase (IDH) were as previously described [7]. The specific activities of SDH and aconitases were reported to the specific activity of IDH. Three independent measurements were performed on four hFXN clones, five hFXN^ G130V^ and three hFXN^ I154F^ mutant clones. Nth1 activity was assessed as described [8]. The IPR1 IRE-binding activities were assessed by EMSA as previously described [55] with some modifications as described in Text S1. The activity of catalase was determined spectrophotometrically as described [56] by measuring the absorbance decrease at 240 nm in a 20 mM phosphate buffer pH 7 containing 10 mM of hydrogen peroxide. The glutathione reductase activity assays were carried out in phosphate buffer (pH 7.4; 0.1 M) which contains 0.5 mM EDTA and 1 mM of oxidized glutathione at 37°C. The reaction was initiated by adding 0.2 mM of NADPH (SIGMA). Activity was determined by following the disappearance of NADPH at 340 nm.

### Iron measurements

Mitochondrial-enriched extracts were mixed with 0.3 volume of HCl 12N and incubated at 40°C for 1 hour. Distilled water was added and precipitated material was removed by centrifugation at 15,000 g, 10 min. The samples were diluted with Tris-base 1 M pH 8.8 and incubated in presence of ascorbic acid 0.25% and bathophenanthroline disulfonic acid 0.02% (Sigma) for 1 hour in the dark. The absorbance at 535 nm of the iron/bathophenanthroline disulfonate complex was measured against a blank containing mitochondrial extract, buffer and reagents without bathophenanthroline. The iron concentration was determined using a molar extinction coefficient of 22,140 M^−1^.cm^−1^ for the complex [57]. For the atomic absorption spectroscopy, mitochondria-enriched fractions were lysed with Tris 0.1 M pH 7.5, Triton X-100 0.5% and mitochondrial soluble fraction was separated from the membrane insoluble pellet by centrifugation 12,000 g, 10 min at 4°C. Supernatant were diluted in pure water (18 MΩ) containing 0.2% nitric acid. The pellets were dissolved in 1 ml of pure nitric acid. The calibration of the iron concentration was done by using standard iron solutions for atomic absorption purchased from Fluka. The graphite furnace used was a Perkin-Elmer 4110ZL spectrometer equipped with an autosampler AS-72.

### Reactive Oxygen Species (ROS) determination

The presence of cellular ROS was assessed by using the redox sensitive probe DiHydroRhodamine 123 (DHR123, Molecular Probes). Fibroblasts (60–80% of confluency) were incubated with 30 µM of DHR123 at 37°C for 15 min, washed twice with PBS and incubated 30 min at 37°C in PBS supplemented or not by 10 µM of hydrogen peroxide. Cells were then treated with trypsin, centrifuged at 1000 g, 5 min in culture medium and washed with PBS. The fluorescence intensity of cells was analysed by flow cytometric analysis in a FACScalibur (Becton-Dickinson; FL-1, band pass filter 530/30 nm). Data were analysed by Flowjo.

### Statistics

All data represent mean±SD. Statistical analyses were performed by standard Student t-test. Statistical significance was considered at p<0.05.

## Supporting Information

Text S1 (0.04 MB DOC)

Figure S1 Murine frataxin protein level in FrdaL-/L- transfected clones. Frataxin protein level in transfected clones. Western Blot analysis of total protein extracts from FrdaL3/L- non-transfected cell line (NT), the FrdaL3/L-; empty growing clone F8, and four FrdaL3/L-; mFxn clones (B6, H5, C10 and E2) using a polyclonal frataxin antibody. β-tubulin detection was used to assess equal loading in each lane. Note that in the F8 clone, we detect endogenous frataxin confirming its non-deleted status.
(1.72 MB TIF)

Figure S2 Morphology of frataxin deficient clones by electron microscopy. Frataxin deleted cells show very little ultrastructural changes. Apart from the retraction of the plasma membrane, no structural anomaly or sign of necrosis or apoptosis was observed in FrdaL3/L-; empty clone (C and D) compared to FrdaL3/L-; mFxn clone (A and B) after six days of culture. No mitochondrial dense material suggestive of iron deposit has been observed. Lp, lipid droplet; mt, mitochondria; N, nucleus; nu, nucleole.
(1.05 MB PDF)

Figure S3 Ultrastructural alterations in hFXNG130V and hFXNI154F clones. Electron microscopy on hFXN (A), hFXNI154F (B) and hFXNG130V (C,D) clones. b, plasma membrane blebbing; g-mt, giant mitochondria; Lp, lipid droplet; mt, mitochondria; mt-Fe, intramitochondrial iron deposits; N, nucleus; os-mt, onion-shaped mitochondria; p, pseudopodia; rer, rough endoplasmic reticulum.
(1.76 MB TIF)

Figure S4 Iron content of hFXN, hFXNG130V and hFXNI154F clones determined by atomic absorption spectroscopy. Mitochondrial soluble fraction or insoluble membrane pellet iron contents were assessed by atomic absorption spectroscopy as described in material and methods. Results are given as mean of µg of iron per mg of protein in each fraction + SD. * p<0.05.
(9.44 MB TIF)
