# Supplementary material for: The First Cellular Models Based on Frataxin Missense Mutations That Reproduce Spontaneously the Defects Associated with Friedreich Ataxia
Source: PLoS One. 2009 Jul 24;4(7):e6379. doi: 10.1371/journal.pone.0006379 (PMC2710521; doi:10.1371/journal.pone.0006379)
Supplement: Text S1 — (0.04 MB DOC) [file pone.0006379.s001.doc]

**Text S1:**

**Supplemental Materials and Methods**

***Primers for quantitative RT-PCR***

Amplification of a 199-bp human frataxin-specific exon 3-5 fragment was carried out using primers hFXN-ex3F: agaggaaacgctggactctt and hFXN-ex4/5R: ACGCTTAGGTCCACTGGATG. For catalase expression, the following primers were used: Cat-F: AGAGGAAACGCCTGTGTGAG and Cat-R: GGCTTCTCAGCGTTGTACTTG.

***IRP1 binding activities***

The IPR1 IRE-binding activities were assessed by EMSA as previously described [1] with some modifications. Briefly, the murine H ferritin IRE was cloned into pcDNA 3.1/Zeo using *NheI* and *HindIII* restrictions sites. The resulting plasmid was used for *in vitro* transcription using [α ^32^P] CTP and the T7 RNA polymerase transcription kit (Promega) according to the manufacturer’s protocol. Cells were lysed in 10 mM HEPES pH7.5, 3 mM MgSO_4_, 40 mM KCl, 2 mM DTT, 0.2 % IGEPAL, Complete 1X (Roche), 4 mg/mL Pefabloc SC, 8 mM βglycerophosphate and 1 mM deferiprone. Protein extracts were incubated with the radioactive IRE-probe [1] in the same buffer without IGEPAL. The gel was exposed on a phospho screen and analysed on a Typhoon 8600 appartus (Molecular Dynamics) for quantification.

**Supplemental references :**

1. Kim S, Ponka P (1999) Control of transferrin receptor expression via nitric oxide-mediated modulation of iron-regulatory protein 2. J Biol Chem 274: 33035-33042.

**Supplemental Figure Legends :**

**Fig. S1: Murine frataxin protein level in Frda^L-/L-^ transfected clones**

Frataxin protein level in transfected clones. Western Blot analysis of total protein extracts from Frda^L3/L-^ non-transfected cell line (NT), the Frda^L3/L-; empty^ growing clone F8, and four Frda^L3/L-; mFxn^ clones (B6, H5, C10 and E2) using a polyclonal frataxin antibody. β-tubulin detection was used to assess equal loading in each lane. Note that in the F8 clone, we detect endogenous frataxin confirming its non-deleted status.

**Fig. S2: Morphology of frataxin deficient clones by electron microscopy**

Frataxin deleted cells show very little ultrastructural changes. Apart from the retraction of the plasma membrane, no structural anomaly or sign of necrosis or apoptosis was observed in Frda^L3/L-; empty^ clone (C and D) compared to Frda^L3/L-; mFxn^ clone (A and B) after six days of culture. No mitochondrial dense material suggestive of iron deposit has been observed. Lp, lipid droplet; mt, mitochondria; N, nucleus; nu, nucleole

**Fig. S3: Ultrastructural alterations in hFXN^G130V^ and hFXN^I154F^ clones.**

Electron microscopy on hFXN (A), hFXN^I154F^ (B) and hFXN^G130V^ (C,D) clones. b, plasma membrane blebbing; g-mt, giant mitochondria; Lp, lipid droplet; mt, mitochondria; mt-Fe, intramitochondrial iron deposits; N, nucleus; os-mt, onion-shaped mitochondria; p, pseudopodia; rer, rough endoplasmic reticulum.

**Fig. S4: Iron content of hFXN, hFXN^G130V^ and hFXN^I154F^ clones determined by atomic absorption spectroscopy**

Mitochondrial soluble fraction or insoluble membrane pellet iron contents were assessed by atomic absorption spectroscopy as described in material and methods. Results are given as mean of µg of iron per mg of protein in each fraction + SD. * p<0.05.
