# Supplementary figures and images for: The First Cellular Models Based on Frataxin Missense Mutations That Reproduce Spontaneously the Defects Associated with Friedreich Ataxia
Source: PLoS One. 2009 Jul 24;4(7):e6379. doi: 10.1371/journal.pone.0006379 (PMC2710521; doi:10.1371/journal.pone.0006379)

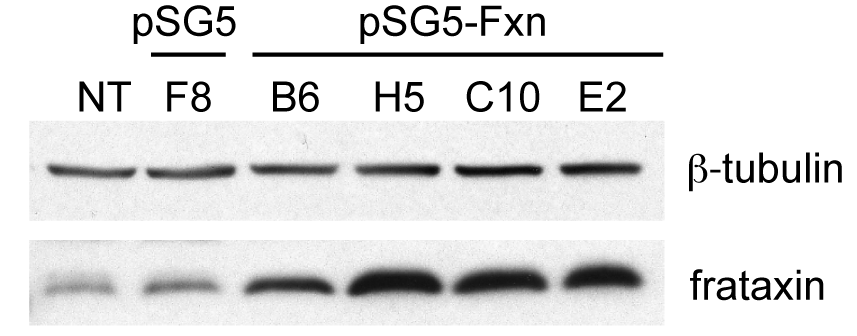

Supplement: Figure S1 — Murine frataxin protein level in FrdaL-/L- transfected clones. Frataxin protein level in transfected clones. Western Blot analysis of total protein extracts from FrdaL3/L- non-transfected cell line (NT), the FrdaL3/L-; empty growing clone F8, and four FrdaL3/L-; mFxn clones (B6, H5, C10 and E2) using a polyclonal frataxin antibody. β-tubulin detection was used to assess equal loading in each lane. Note that in the F8 clone, we detect endogenous frataxin confirming its non-deleted status. (1.72 MB TIF) [file pone.0006379.s002.tif]

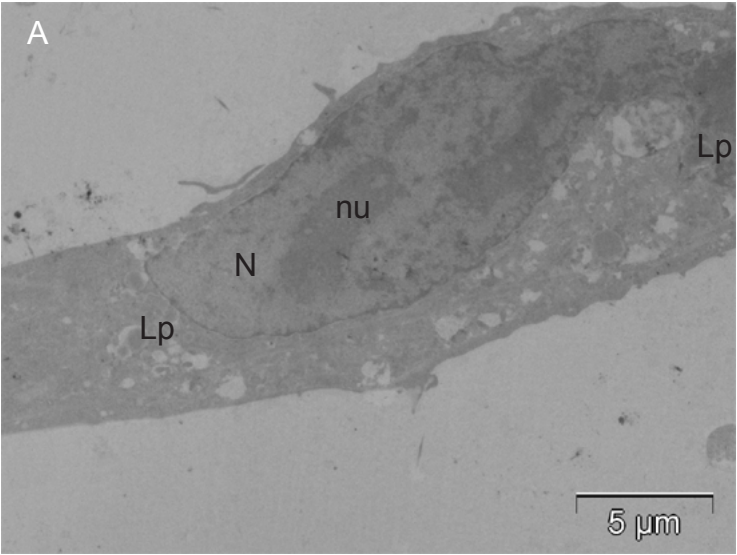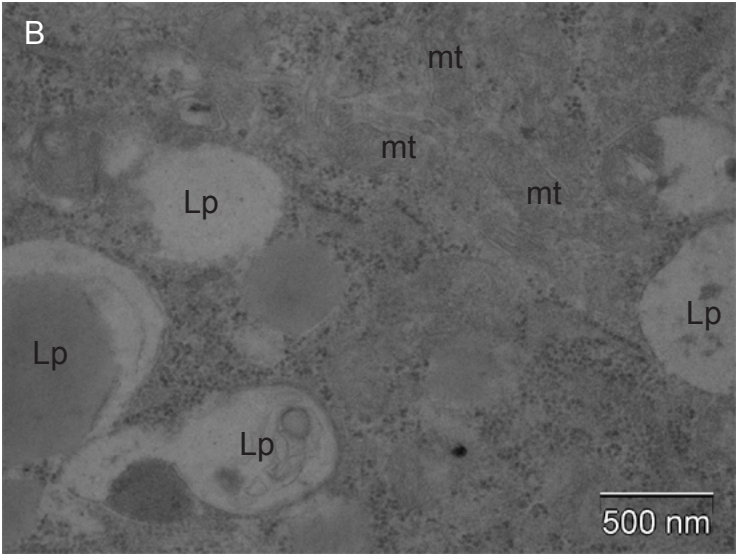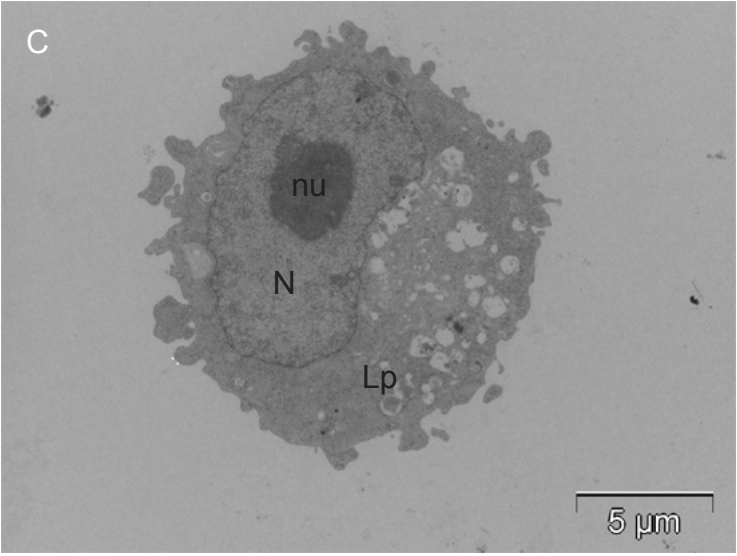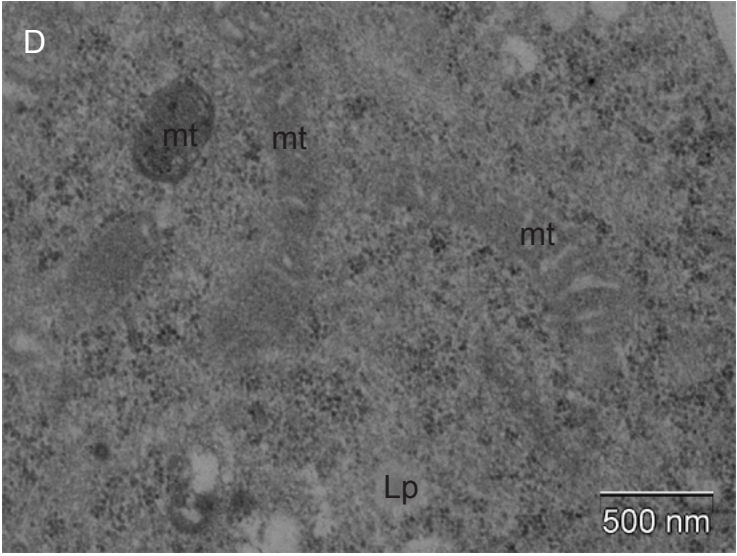

Supplement: Figure S2 — Morphology of frataxin deficient clones by electron microscopy. Frataxin deleted cells show very little ultrastructural changes. Apart from the retraction of the plasma membrane, no structural anomaly or sign of necrosis or apoptosis was observed in FrdaL3/L-; empty clone (C and D) compared to FrdaL3/L-; mFxn clone (A and B) after six days of culture. No mitochondrial dense material suggestive of iron deposit has been observed. Lp, lipid droplet; mt, mitochondria; N, nucleus; nu, nucleole. (1.05 MB PDF) [file pone.0006379.s003.pdf]

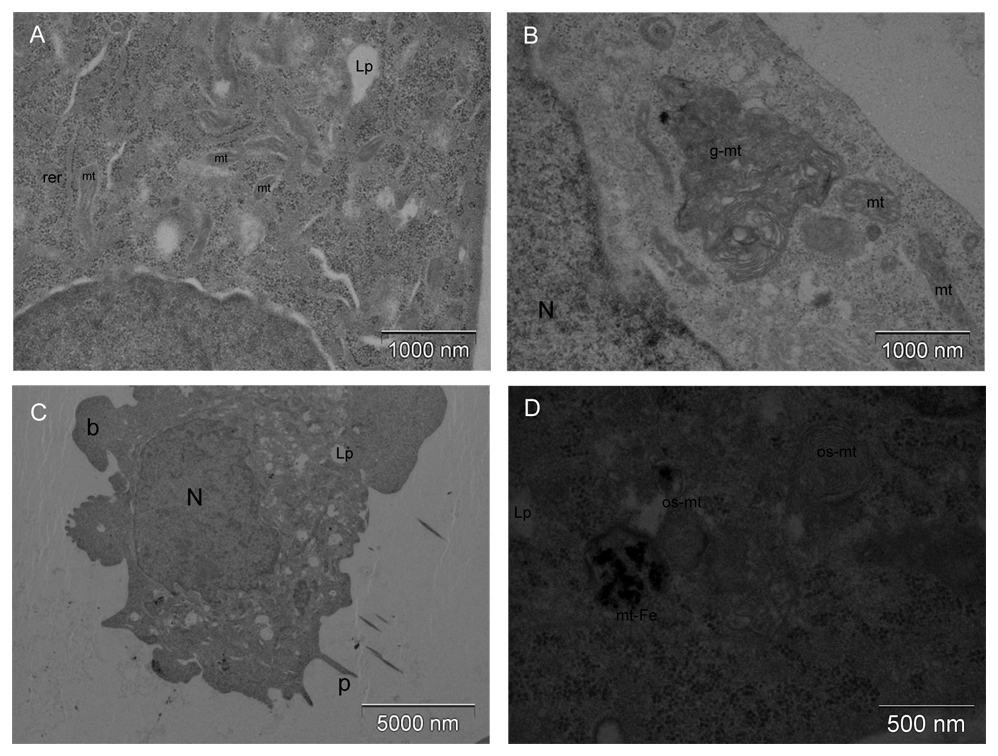

Supplement: Figure S3 — Ultrastructural alterations in hFXNG130V and hFXNI154F clones. Electron microscopy on hFXN (A), hFXNI154F (B) and hFXNG130V (C,D) clones. b, plasma membrane blebbing; g-mt, giant mitochondria; Lp, lipid droplet; mt, mitochondria; mt-Fe, intramitochondrial iron deposits; N, nucleus; os-mt, onion-shaped mitochondria; p, pseudopodia; rer, rough endoplasmic reticulum. (1.76 MB TIF) [file pone.0006379.s004.tif]

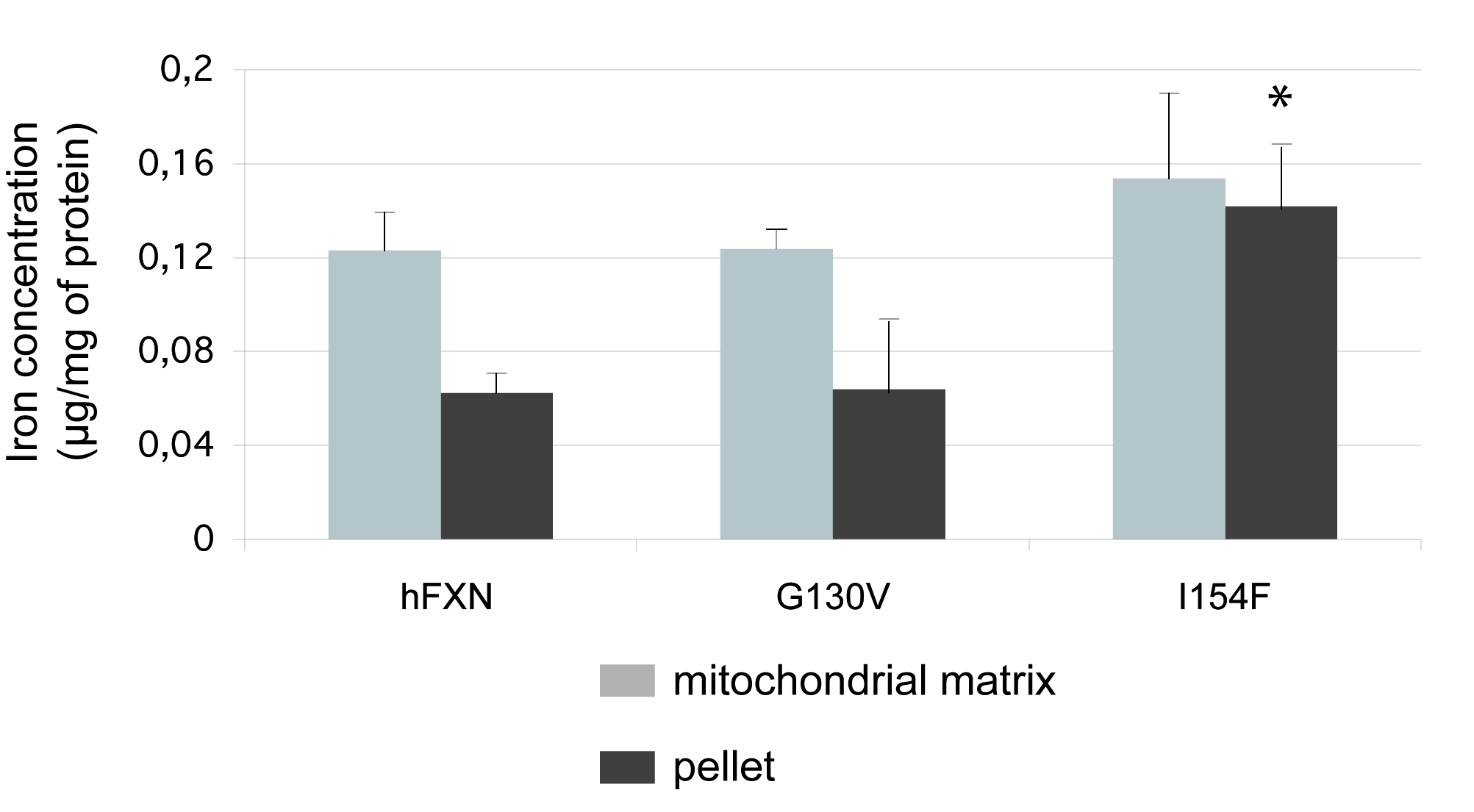

Supplement: Figure S4 — Iron content of hFXN, hFXNG130V and hFXNI154F clones determined by atomic absorption spectroscopy. Mitochondrial soluble fraction or insoluble membrane pellet iron contents were assessed by atomic absorption spectroscopy as described in material and methods. Results are given as mean of µg of iron per mg of protein in each fraction + SD. * p<0.05. (9.44 MB TIF) [file pone.0006379.s005.tif]
